# Effects of marker density and population structure on the genomic prediction accuracy for growth trait in Pacific white shrimp *Litopenaeus vannamei*

**DOI:** 10.1186/s12863-017-0507-5

**Published:** 2017-05-17

**Authors:** Quanchao Wang, Yang Yu, Jianbo Yuan, Xiaojun Zhang, Hao Huang, Fuhua Li, Jianhai Xiang

**Affiliations:** 1 Key Laboratory of Experimental Marine Biology, Institute of Oceanology, Chinese Academy of Sciences, Qingdao, 266071 China; 2 University of Chinese Academy of Sciences, Beijing, 100049 China; 3 Hainan Guangtai Ocean Breeding Co., LTD, Wenchang, 571300 China; 4 Laboratory for Marine Biology and Biotechnology, Qingdao National Laboratory for Marine Science and Technology, Qingdao, China

**Keywords:** Heritability, Genomic selection, Growth traits, Penaeid shrimp

## Abstract

**Background:**

Due to the great advantages in selection accuracy and efficiency, genomic selection (GS) has been widely studied in livestock, crop and aquatic animals. Our previous study based on one full-sib family of *Litopenaeus vannamei* (*L. vannamei*) showed that GS was feasible in penaeid shrimp. However, the applicability of GS might be influenced by many factors including heritability, marker density and population structure etc. Therefore it is necessary to evaluate the major factors affecting the prediction ability of GS in shrimp. The aim of this study was to evaluate the factors influencing the GS accuracy for growth traits in *L. vannamei*. Genotype and phenotype data of 200 individuals from 13 full-sib families were used for this analysis.

**Results:**

In the present study, the heritability of growth traits in *L. vannamei* was estimated firstly based on the full set of markers (23 K). It was 0.321 for body weight and 0.452 for body length. The estimated heritability increased rapidly with the increase of the marker density from 0.05 K to 3.2 K, and then it tended to be stable for both traits. For genomic prediction on the growth traits in *L. vannamei*, three statistic models (RR-BLUP, BayesA and Bayesian LASSO) showed similar performance for the prediction accuracy of genomic estimated breeding value (GEBV). The prediction accuracy was improved with the increasing of marker density. However, the marker density would bring a weak effect on the prediction accuracy after the marker number reached 3.2 K. In addition, the genetic relationship between reference and validation population could influence the GS accuracy significantly. A distant genetic relationship between reference and validation population resulted in a poor performance of genomic prediction for growth traits in *L. vannamei*.

**Conclusions:**

For the growth traits with moderate or high heritability, such as body weight and body length, the number of about 3.2 K SNPs distributed evenly along the genome was able to satisfy the need for accurate GS prediction in the investigated *L.vannamei* population. The genetic relationship between the reference population and the validation population showed significant effects on the accuracy for genomic prediction. Therefore it is very important to optimize the design of the reference population when applying GS to shrimp breeding.

## Background

Selective breeding is recognized as a main propelling force for the development of efficient and sustainable aquaculture production [1]. For many years, mass selection is the most commonly used method for selection breeding in aquatic species because it is easy to manipulate. Although mass selection is a practical approach for the traits that can be recorded without damage [2], family-based selective breeding method has become the industry standard in aquatic species due to the advantage that it is effective for all types of traits such as carcass quality or disease resistance [1]. Several family-based breeding programs, aiming to improve growth and disease resistance traits in *Litopenaeus vannamei* (*L. vannamei*), have been performed in a number of countries since 1993 [3–6]. These programs have improved the target traits a lot and made great contributions to the development of shrimp industry. To date, the yields of *L. vannamei* contributes approximately 42% of the global shrimp production, so this species is regarded as one of the most important cultured shrimp species in the world [7]. However, due to the difficulties manifested for family selected breeding, such as the high investment, high operational costs [8], limitation in the capacity of tested families and low correlation among multiple traits [1], the strains with multiple superior traits, such as high growth rate and disease resistance/tolerance are seldom reared through traditional genetic selection in penaeid shrimp. Therefore, new methods and technologies are urgently required for accelerating the genetic improvement of important traits in *L. vannamei*.

MAS has become a promising method to speed up the selective progress in both plants and animals. The development of molecular markers in *L. vannamei* accelerated the MAS for the interesting economic traits [9–11]. Detection on the quantitative trait loci (QTLs) associated with growth traits such as body weight and body length in *L. vannamei* have been performed, and several significant QTLs for growth traits were revealed [12]. However, these QTLs can explain only a small part of phenotypic variation. More likely, the growth of *L. vannamei* is controlled by multiple QTLs with small effects, so the traditional MAS tend to be inefficient. Alternatively, genomic selection (GS), one kind of MAS which can use genome-wide markers to calculate genomic estimated breeding value (GEBV) for candidate breeding animals [13, 14], has been regard as a powerful method for genetic improvement of complex traits, such as complex quantitative traits, carcass quality traits, and disease resistance traits etc. [15]. GS has been successfully implemented in livestock and plant [16–18]. Compared with the traditional breeding method, GS has great advantage in selection accuracy, and more importantly it can greatly accelerate the breeding process and reduce the costs of a breeding project since selection can be carried out at an early growth stage without the need for phenotypic measurements [19–21]. Although GS is a relatively new approach for aquatic breeding, it is now getting more attention from aquatic breeders, and a series of excellent work for the application of GS have been performed. The possible strategies to implement genomic selection in aquaculture breeding schemes were evaluated based on the simulated data, and the results showed that GS could generate high genetic gain, high selection accuracy and low inbreeding rates under the current family-based breeding schemes [22]. More recently, by using real data, a few studies in several aquatic species have been carried out to investigate the applicability of GS. The GS method outperformed the classical pedigree-based selection for growth traits and disease resistance traits in Atlantic salmon and gilthead sea bream [23–25]. The performances of GS were varied in these species because of the differences in genome size and the progresses in breeding [24–26]. Therefore, it is important to evaluate the performance of GS with respect to shrimp.

In a previous study, we found that GS was feasible for growth traits in *L. vannamei* based on one full-sib family [27]. For within-family genomic selection, low-density markers are required to perform genomic prediction due to the small effective population size [28]. However, in practice, a large number of families are usually produced in family-based breeding programs. Under this situation, high-density markers are necessary for the increasing effective population size. Moreover, the broad-based population might lead to population stratification at some extent, which will influence the accuracy of GS. Therefore, a detailed survey of GS based on multiple families is necessary for *L. vannamei*.

In the present study, all samples from 13 families, which derived from different commercial lines were used. Therefore, it is a good material to estimate the impact of potential factors on the accuracy of GS in applied breeding programs. This study will be helpful for the further application of GS in *L. vannamei*.

## Methods

### Animals

All samples used in this study were from Guangtai Marine Breeding Company in Hainan province, China. Totally, 13 full-sib families (offsprings of 13 dams and 13 sires) were created in July 2015. Each full-sib family was cultured separately in the 5 m^2^ tank for seedlings. After grown to 3 cm, 50 individuals from each family were transferred to a 10 m^2^ pond for culture. At the harvest, two hundred individuals were randomly collected, and two growth traits, including body weight and body length, were measured.

### Genotyping and quality control

Genomic DNA of each sample was isolated from muscles of shrimp using Plant Genomic DNA Kit (TIANGEN, Beijing, China) following the manual instruction. The purity and integrity of extracted DNA was determined by using a NanoDrop 1000 Spectrophotometer (NanoDrop, Wilmington, DE, USA) and electrophoresis on 1% agarose gel. Qualified genomic DNA was stored at −20 °C.

All individuals were genotyped using 2b–RAD method which was performed by Oebiotech Company (Oebiotech, Shanghai, China). Briefly, the 2b–RAD libraries were prepared following the standard protocol [29], and then they were pooled for sequencing using Illumina HiSeq X Ten platform. The genotyping was performed using the RADtyping program v1.537 with default parameters [30].

The quality control of SNP data was performed using R software [31]. SNPs with missing rate across samples more than 5% and minor allele frequency less than 0.05 were removed. After quality control, a total of 23,049 SNPs were obtained. Furthermore, the missing SNPs were imputed using Beagle 3.3.2 with default parameter settings [32].

### Estimation of the heritability

In this study, the narrow-sense heritability (h^2^) of each trait was defined as the ratio of additive genetic variance to the total phenotypic variance (*V*_A_/*V*_P_), and was estimated using the genetic relationship matrix calculated based on genetic markers. The variance components were estimated using the package rrBLUP [33]. Firstly, the marker-based additive relationship matrix (G matrix) for the 23,049 (23 K) markers was calculated using the *A.mat* function in rrBLUP package with the default options. Then, the *kin.blup* function, taking G matrix as covariance matrix, was used to estimate the variance components. Additionally, 10 Random-distributed marker subsets (0.05 k, 0.1 K, 0.2 K, 0.4 K, 0.8 K, 1.6 K, 3.2 K, 6.4 K, 12.8 K, and 20 K) were used to investigate the impact of marker density on heritability estimation. To reduce the sampling error, 50 random selections of SNPs for each subset were chosen from the full marker set according to the subset size. The heritability was calculated by averaging the results of the 50 random selections for each marker subset. The method for the heritability estimation based on each marker subset was the same as that calculated using the full set of markers.

### Population stratification assessment

A multidimensional scaling (MDS) analysis was performed using the *cmdscale* function in R software [31] to verify the genetic homogeneity of the dataset. Firstly, the matrix of genomic kinship (Identity By State) was calculated based on the filtered markers with the GenABEL package [34]. Then the matrix of genomic kinship was inputted into R software [31] and the MDS was calculated using “*cmdscale*” function. In addition, samples were divided into several clusters (subpopulation) using the k-means method in R software [31].

### Statistical models

Three statistical models were used to predict the genomic estimated breeding value (GEBV) for two growth traits: ridge regression best linear unbiased prediction (RR-BLUP), Bayesian LASSO (BL), and BayesA (BA). All models estimated the marker effects by incorporating the markers as random effects, and no fixed effects were fitted in the models. The RR-BLUP model was fitted by the R package rrBLUP [33], and the BL and BA models were implemented by the R package BGLR [35] with the default parameters. For the BL and BA models, the Gibbs sampler was run for 40,000 iterations, with the first 10,000 iterations discarded as burn-in. After the marker effects were estimated by the models, the GEBV of individuals was computed to allow for the validation of populations, given by Eq. (1):

1 $$ GEBV= Xg $$

where X is the corresponding design matrix with elements of X_ij_ = 0, 1, 2 for genotypes AA, AB and BB, respectively for the i th animals and j th SNP; g is the vector of additive effects of markers estimated by the models.

### Cross-validation

The accuracy of genomic prediction was measured as the correlation between the GEBV of shrimp in validation population and their observed phenotypes divided by the square root of trait heritability (estimated using full marker). In order to assess the impact of several factors on the accuracy of genomic prediction, different cross-validation approaches were used.

To determine the effect of marker density on the prediction accuracy, a five-fold cross-validation approach was carried out [36]. In brief, the samples were randomly divided into five subsets, each containing 20% of data. For each cross-validation experiment, one of the five subsets was retained as the validation set, and the other four subsets combined and served as the training set. The process was repeated five times, each time with one subset as validation set. Accordingly, each individual appeared only once in the validation set and had only one predicted GEBV. Random-distributed marker subsets of different sizes were selected and used for comparison with the full marker set [36]. The marker density was varied in each subset as follows: 0.05 k, 0.1 K, 0.2 K, 0.4 K, 0.8 K, 1.6 K, 3.2 K, 6.4 K, 12.8 K, and 20 K. For each subset, 50 random selections of SNPs were chosen from the full marker set according to the subset size. Then, each selection for each marker subset was used as the genotype matrix to perform five-fold cross-validation. The accuracy for each marker density was calculated by averaging the cross-validation results of 50 random selections. In addition, the full 23,049 (23 K) SNP set was also used to perform five-fold cross-validation as a reference.

**Fig. 1:**
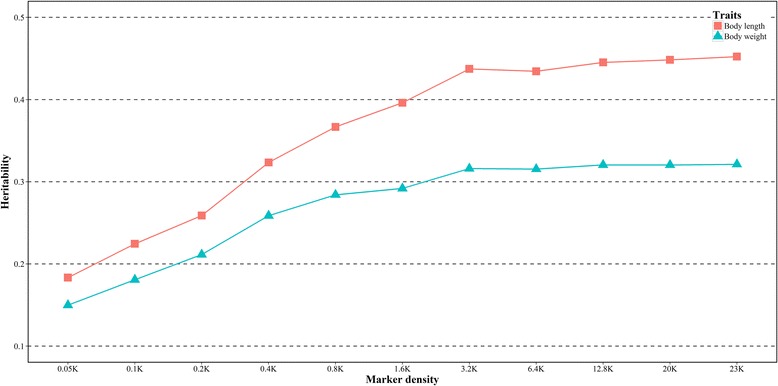
The estimated heritability of body weight and body length with varied marker densities

To investigate the impact of the genetic relatedness between reference and validation population on prediction accuracy [37], two groups (DIST and RAND) were created based on the detected genetic relationships shown in Fig. 1. The DIST group contained three subgroups (DIST1, DIST2 and DIST3), in which the genetic relationship between the reference and validation population was distant. The validation sets for DIST1, DIST2 and DIST3 were the subpopulation IV, V and VI (size >30) discovered by k-means analysis and the remaining subpopulations were combined as the training set respectively. The RAND group also contained three subgroups (RAND1, RAND2 and RAND3), in which the genetic relationship between the reference and validation population was close. For each of the three RAND subgroups, a random sample of 80% from each subpopulation was selected, then combined and served as the training set, and the remaining samples of each subpopulation were combined and served as the validation set. These analyses were performed with all 23,049 (23 K) SNPs.

## Results

### Information of the phenotypes

The phenotypic statistics for body weight and body length were given in Table 1. The average body weight and body length of the individuals used for the phenotype analyses were 5.56 ± 2.16 g and 76.99 ± 9.95 mm separately.

**Table 1:** Mean, minimum, maximum and standard deviation (SD) for body weight and body length

	Mean	Minimum	Maximum	SD
Body Weight (g)	5.56	1.32	12.13	2.16
Body Length (mm)	76.99	48.80	100.65	9.95

### Estimation of the heritability for growth traits

The estimated heritability was shown in Fig. 1. It was 0.321 and 0.452 for body weight and body length, respectively, based on the full set of markers (23 K). With the increase of the marker density, the estimation value of heritability tended to be raised until reaching a certain amount of markers. For body weight, the heritability was ranged from 0.150 to 0.321. The estimated heritability of body length showed similar tendency, and it was ranged from 0.183 to 0.452. Interestingly, the estimated heritability increased rapidly with the increase of marker density from 0.05 K to 3.2 K, and then the value tended to be stable for both traits.

### Population stratification

**Fig. 2:**
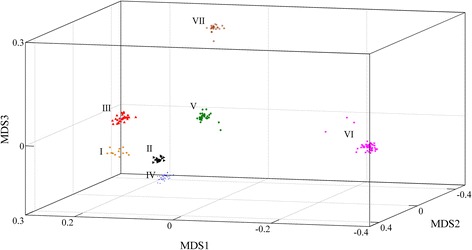
Multidimensional scaling (MDS) analysis of an Identity By State (IBS) matrix for 200 samples

The multidimensional scaling (MDS) analysis of an Identity By State (IBS) matrix for 200 samples was shown in Fig. 2. Based on the first three principal components of MDS analysis, all samples were clustered into seven subpopulations (I, II, III, IV, V, VI and VII). The largest subpopulation was VI, followed by V, IV, III, VII, II and I, including 52, 35, 32, 28, 23, 18 and 12 individuals respectively.

### Accuracy of genomic prediction with different marker density

**Fig. 3:**
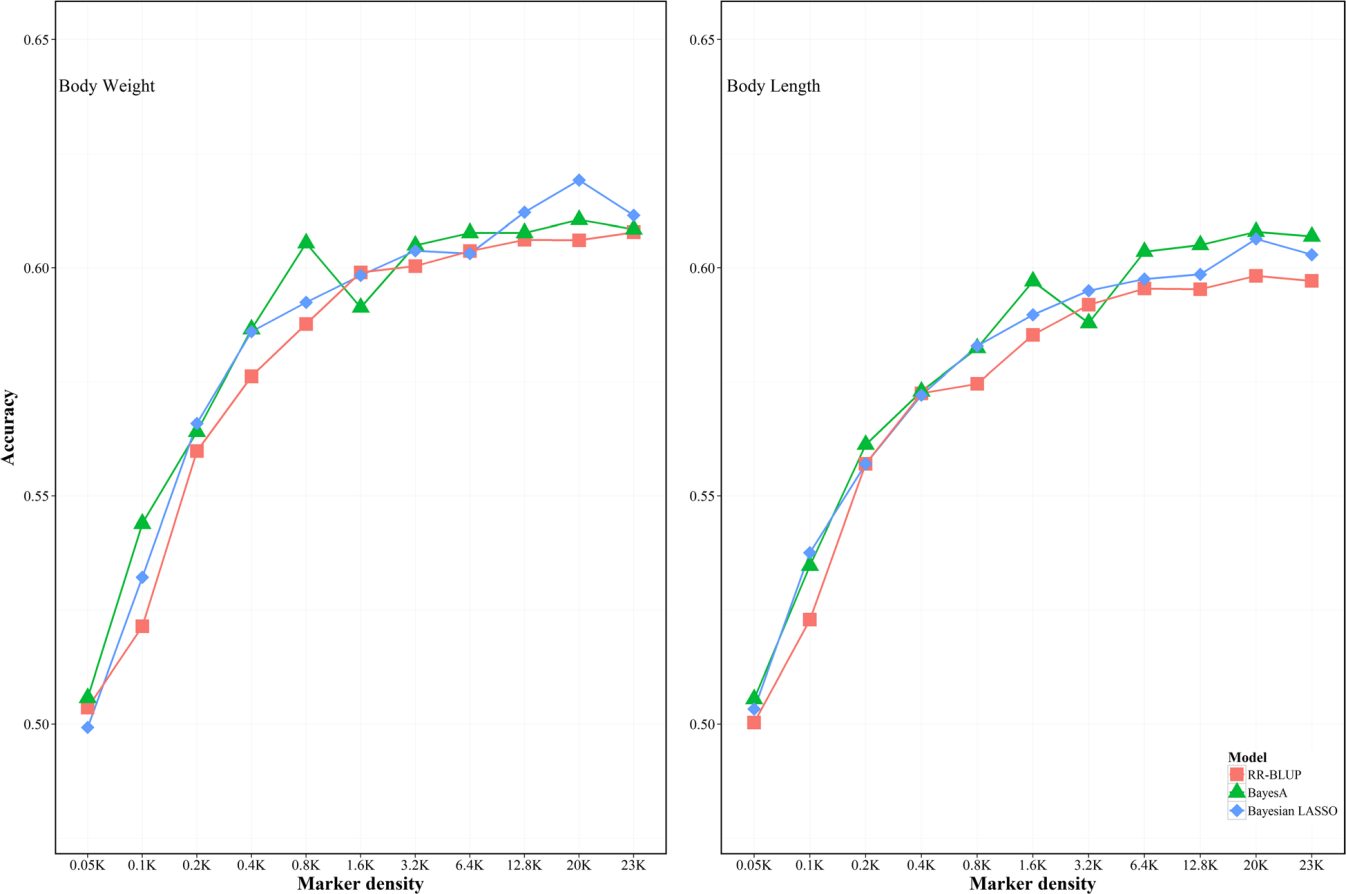
Mean accuracies of cross-validation for genomic prediction of body weight and body length predicted by three statistical models under different marker densities

The average prediction accuracy for body weight and body length with different marker density was shown in Fig. 3. The mean accuracy ranged from 0.499 to 0.619 for body weight and from 0.500 to 0.607 for body length. For body weight, Bayesian LASSO (BL) performed slightly better than RR-BLUP and BayesA (BA) when evaluated with marker densities from 12.8 K to 23 K. In contrast, BA showed a better performance than RR-BLUP and BL for body length when the number of markers was more than 3.2 K. In addition, the prediction accuracy increased rapidly with the increase of marker density from 0.05 K to 3.2 K, and then there was very little improvement in prediction accuracy when the marker density kept to increase.

### Accuracy of genomic selection with different reference sets

**Fig. 4:**
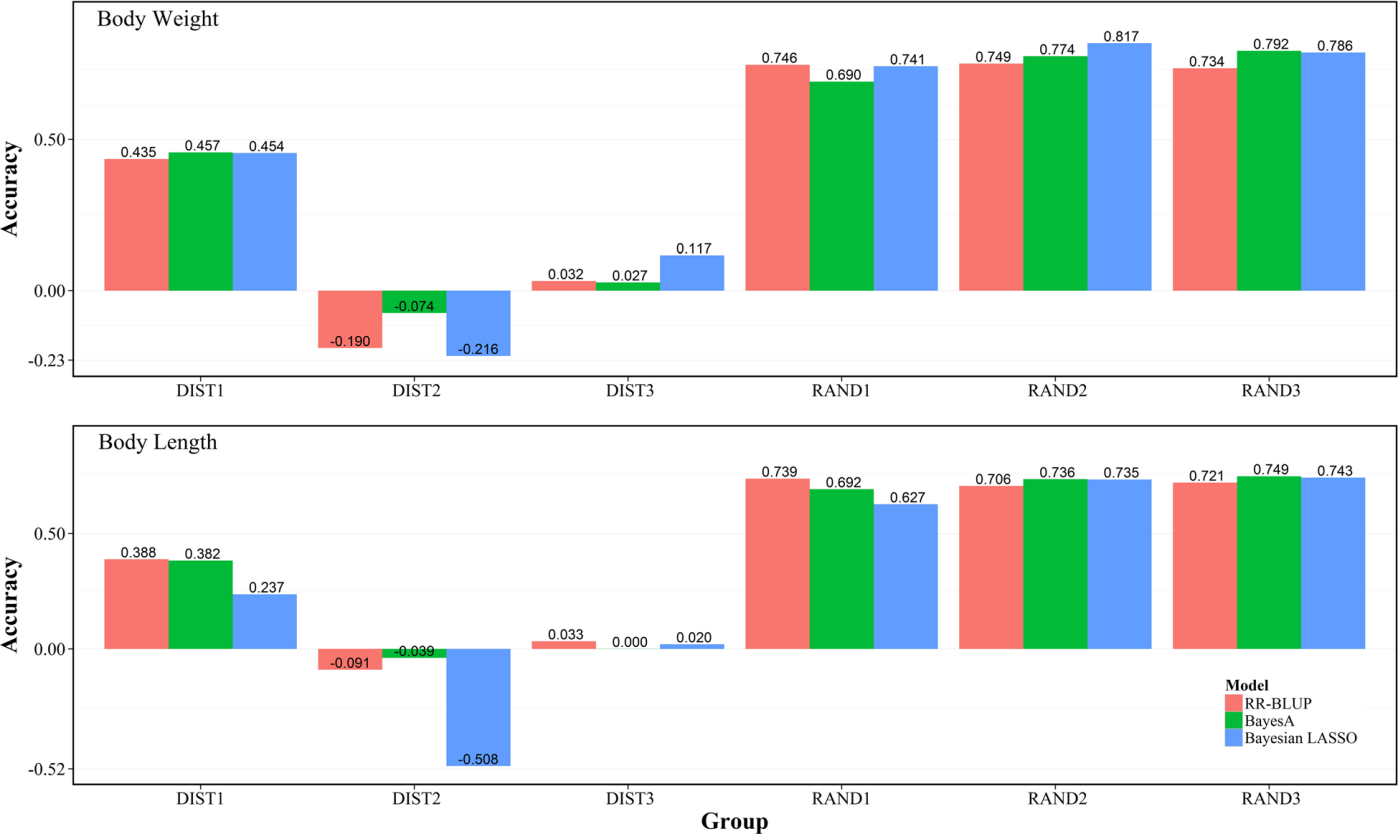
Accuracies of cross-validation for genomic prediction of body weight and body length using a full marker set for DIST group (DIST1, DIST2 and DIST3) and RAND group (RAND1, RAND2, RAND3)

Across the growth traits, the RAND group showed higher prediction accuracy than the DIST group (Fig. 4). For body weight, the mean accuracy estimated by RR-BLUP, BA and BL for the RAND group was 0.743, 0.752 and 0.781 respectively, which was about sevenfold higher than that for the DIST group; the highest accuracy, predicted by BL for RAND2, was up to 0.817, and the lowest accuracy was −0.216 predicted by BL for DIST2. Similar results were also obtained for body length. In the RAND group, the mean accuracies produced by RR-BLUP, BA and BL were 0.722, 0.726 and 0.701 respectively, while in the DIST group, the mean accuracies produced by RR-BLUP, BA and BL were 0.110, 0.114 and −0.08 respectively. The highest accuracy, predicted by BA for RAND3, was up to 0.749, and the lowest accuracy was −0.508 predicted by BL for DIST2.

## Discussion

### Heritability estimation by SNP markers

Narrow-sense heritability is a central parameter in quantitative genetics and represents the proportion of total phenotypic variance that is due to additive genetic effects. Traditionally, the heritability of growth traits in *L. vannamei* was estimated by using pedigree information, which is time-consuming and expensive [3, 5, 38–40]. To our knowledge, the current study is the first report for heritability estimation in *L. vannamei* by using genome-wide markers. The estimated heritability based on the full-set of genome-wide markers was 0.321 for body weight and 0.452 for body length. This value estimated in this study was located at the reported range of 0.24–0.515 estimated based on pedigree information for growth traits in *L. vannamei* [5, 41–44]. However, the estimated heritability for body weight in the present study was significantly lower than those reported by Argue et al. [3] from 0.71 to 0.84. The different heritability values for the same trait among these studies may be caused by different estimation methods, different numbers of families, environmental interactions and unpredictable genetic effects [39]. From the present data, we can conclude that the marker density in the genome was one major factor affecting the estimated accuracy of heritability for growth traits. The minimum of 3.2 K SNPs might be the baseline for heritability estimation within this population of *L. vannamei*.

### Statistical models for GEBV prediction

There are already some reports to evaluate the performance of various GS models [45–47]. In the simulated studies based on the assumption that the traits are controlled by a limited number of QTLs with large-effects, Bayesian models (e.g., Bayesian regressions and LASSO) always showed superior performance on prediction accuracy [48]. However, only small differences in accuracies were observed between statistical models for empirical evaluation. For some traits controlled by no known genes with large effects, BLUP-based models can produce similar or superior accuracy than Bayesian models [49, 50]. In the present study, three statistical models showed similar prediction ability for GEBV, which was the same as our previous report based on single-family shrimp population [27]. Therefore, it might give us a hint that the growth traits of *L. vannamei* might be controlled by a large number of QTLs with small effects.

### Effects of marker density on GS

An increase of marker densities generally resulted in raised accuracy predicted by three statistical models for both body length and body weight. However, above a threshold of approximately 3.2 K, the increase of marker density showed minor effects on the improvement of prediction accuracy. Similar phenomenon was found in other species although the threshold might be different. With a SNP density of around 5 K, the prediction accuracy for host resistance to sea lice in farmed Atlantic salmon reached a plateau [24]. For several traits in rice, there was no significant difference in prediction accuracy when 7142 SNPs and 73,147 SNPs were used respectively [36]. The threshold where the plateau takes place might be determined by the extent of linkage disequilibrium (LD) between markers and QTLs in the genome. Theoretically, the extent of LD in a population is related with effective population size (*N*e) [51, 52]. At low *Ne*, the number of independent segments in the genome is expected to be small, and fewer independent segments means that fewer markers are needed to mark all segments [53]. In the present study, the population was considered to have a relatively small effective population size since all samples were from 13 full-sib families, and hence a small number of markers was sufficient to produce the accurate prediction.

### Effect of the genetic relatedness between reference and validation population on GS

The accuracy of genomic prediction can also be affected by the genetic relatedness of the reference and validation population [54, 55]. In order to assess its effect on prediction accuracy of growth traits in *L. vannamei*, we designed two different groups (DIST and RAND) according to the population stratification. As a result, relatively lower accuracy was observed across both traits in DIST group than that in RAND group. It suggested that the poor relatedness between the reference and validation population had negative effect on genomic prediction accuracy. This result was crucial for the future application of GS in shrimp. For selective breeding in shrimp, the core germplasm is generally made up of many families, which easily leads to population stratification. Therefore, the composition of the reference population is very important for the predicted accuracy of GS. The results of the current study also suggested that high accuracy of GS can be achieved by the optimal design of the reference population even if the population stratification exists. To achieve high prediction accuracy of GS, the reference population needs to be more representative for the prediction population in genetic diversity. For GS of shrimp in family-based breeding programs, suitable sampling from different families may be an effective strategy for the design of reference population.

### Implications

The previous studies reported that the performance of genomic prediction may depend on many factors, such as the trait heritability, the genetic architecture, the marker density, the training set size, and the relatedness between the training population and validation population [56–58]. However, in practice, not every factor can be controlled and has same effect on the prediction accuracy. In fact, the heritability and genetic architecture cannot be controlled for specific traits. In contrast, the statistic models, marker density, and the design of reference data sets can be optimized to improve the accuracy of genomic prediction. From the perspective of statistic models, all three models appeared practicable for GS prediction on growth traits with similar prediction ability in *L. vannamei*. Furthermore, it is especially interesting that high prediction accuracy can be obtained with relatively low marker density (3.2 K), which implies that the genomic selection for growth traits of *L. vannamei* could be realized with low costs for marker genotyping. Nevertheless, several factors should be taken into consideration in practical application: firstly, different breeding populations may correspond to different *Ne,* and thus require different number of markers to perform genomic selection; Besides, the expected accuracy of GS will rapidly decay as a result of the decreasing family relationship after several generations of selection. Therefore, higher marker density is expected to capture more LD in the population for multi-generation selection. In addition, although the size of current population is limited, the genetic relationship is complex and its impact on the accuracy of GS is significant. Considering that the shrimp may be from different population in most shrimp breeding projects, the genetic relationship may be more complex than the studied population. Therefore, it is extremely valuable to analyze the population structure firstly and then optimize the design of the reference population to achieve the high accuracy of GS in shrimp breeding program. In genomic selective breeding programs, maximizing the genetic diversity and balancing the contribution of alleles may be an effective strategy for the design of reference population.

## Conclusions

In this study, the heritability estimated using a full set of genome-wide markers was 0.321 and 0.452 for body weight and body length, respectively. The present study showed that genomic selection was an efficient approach in *L. vannamei* breeding programs. All three models appear to be applicable for GS of growth traits in *L. vannamei*. Relatively low marker density (around 3.2 K) was sufficient for accurate prediction on the breeding value for both traits in the studied population. The relatedness between reference and validation set showed a great effect on the prediction accuracy for the growth traits in shrimp. Therefore, it is necessary to analyze the population structure and then optimize the design of the reference population in the future application.

## Abbreviations

BLUP: Best linear unbiased prediction
GEBV: Genomic estimated breeding value
GS: Genomic selection
MAS: Marker assisted selection
IBS: Identity By State
LD: Linkage disequilibrium
MDS: Multidimensional scaling analysis
*N*e: Effective population size
QTL: Quantitative trait loci
RR-BLUP: Random regression–best linear unbiased prediction
SNP: Single nucleotide polymorphism
